# Essential Evidence Plus

**DOI:** 10.5195/jmla.2017.211

**Published:** 2017-07-01

**Authors:** Jessica A. Koos

Essential Evidence Plus (EEP) is a point-of-care tool that was created to assist clinicians with evidence-based practice. According to the creators, the database was designed with the work-flow of health care providers in mind in order to facilitate clinical decision making [1]. It contains information on 9,000 common diagnoses and offers access to over 13,000 individual resources [1]. The intended audience is medical doctors, nurses, and any other health care providers that interact directly with patients [1]. EEP can, therefore, also be a valuable tool for pharmacists, physician assistants, nurse practitioners, and more. In addition, students in the health sciences as well as health sciences librarians can benefit from having access to the information that EEP provides.

The editorial board is composed primarily of physicians [1], and there is a Medical Advisory Board consisting of a large number of physicians from across the United States [2]. Topics in EEP are updated continuously; each topic is fully reviewed three times annually; and drug safety alerts are incorporated within seventy-two hours [1].

## SEARCHING AND CONTENT

EEP consists of several databases that can be searched individually or simultaneously by entering the search terms into a single search box. No advanced search option exists, but Boolean operators can be used. When searching for specific terms across all databases, options are available to narrow the search by content or resource. The results can also be sorted by relevance, date, or title. Descriptions of the individual database options follow.

### Essential Evidence Topics

This database is organized with listings of broad subject areas; selecting one of these reveals more specific subtopics. After selecting a subtopic, users are then presented with an “Overall Bottom Line,” which is a summary of the latest clinical evidence. Each statement is followed by a level-of-evidence grade, which is also a link. Hovering the cursor over the link provides brief information about the evidence rating that was used in creating the grade. Selecting the link leads to a more detailed description of the grading procedure.

There is also more specific information provided on the topic, such as background information, prevention, diagnosis, treatment, and so on. Hovering the cursor over the reference numbers provided after some of the statements reveals the citation of the original study, as well as a link to the article record in PubMed and a Cochrane abstract or relevant Patient-Oriented Evidence that Matters (POEM) research summaries (see below), if available. Some topics include an “Interactive Tools” box, which contains various instruments such as calculators and/or algorithms specific to the topic.

### Cochrane Systematic Reviews

Summaries of Cochrane Systematic Reviews can be searched by entering terms in the search box or by navigating a hierarchy of topics and subtopics similar to those used for Essential Evidence Topics. A link to the full review is provided, but it can only be accessed if the institution or individual has a subscription to Cochrane.

### Patient-Oriented Evidence that Matters (POEM) Research Summaries

One unique feature of EEP is a daily POEM research summary. These brief research study descriptions focus on evidence-based outcomes that are important to patients, such as better quality of life and longer life expectancy, as opposed to other outcomes that are considered less important to patients, such as improved lab values. In addition, POEMs suggest a change in practice for issues regularly encountered in clinical settings [3]. For example, a clinician reads a POEM that provides evidence that a new medication can increase life expectancy for cardiac patients and, in turn, decides to pre-scribe that medication in place of the treatment that the clinician currently renders. Users can register to receive each daily POEM via email. For more in-depth information, weekly podcast discussions of Daily POEMs are also available.

### EBMG Guidelines and EBMG Evidence Summaries

As the title implies, these databases contain evidence-based medicine guidelines and evidence-based summaries, respectively. They can be searched using a search box or browsed by topics and subheadings.

### Decision Support Tools

This area contains calculators and other tools to aid clinicians in making evidence-based treatment decisions based on individual patient factors [1].

### History and Physical Exam Calculators

These calculators facilitate the clinician’s ability to determine a patient’s risk for developing a specific medical condition [1]. Links to original re-search articles in PubMed are provided when available.

### Diagnostic Test Calculators

These calculators can be used by clinicians to interpret lab results and determine which laboratory tests to order [1]. Links to the original research articles in PubMed are provided.

### Derm Expert Image Viewer

This tool provides images of various skin conditions to aid in diagnosis.

### E/M Coding

This resource is designed to facilitate reimbursement from Medicare.

## SPECIAL FEATURES

### Levels of Evidence

EEP enables users to access the original research easily, as well as detailed information regarding the methods used to determine the levels of evidence. These features allow users to review and evaluate the information in EEP for themselves in order to determine if they would like to incorporate it into their practices.

### Drug Safety Alerts

According to EEP, “Within 72 hours of new, serious drug safety concerns or drug withdrawals, the Essential Evidence Topics will be up-dated and an e-mail alerting service will notify users of the latest safety information” [1]. These alerts provide an efficient and effortless way for clinicians to keep up with current drug information and avoid prescribing medications that can cause adverse effects for their patients.

### Continuing Medical Education (CME)

Physicians can receive continuing medical education (CME) credits through conducting specific types of searches using EEP and by providing documentation of the searches.

## PLATFORM

EEP can be accessed using recent versions of Firefox, Chrome, Explorer, and Safari. Institutional access can be provided through Internet protocol (IP) authentication. While a mobile app is not available, EEP offers a mobile-friendly interface when users access the site with mobile devices. One limitation of the mobile version is that the content cannot be downloaded onto mobile devices, so access requires an Internet connection.

## COMPARISON TO OTHER SIMILAR DATABASES

The cost of an individual subscription is significantly less than the cost of other similar point-of-care tools. For example, an individual physician subscription to DynaMed Plus costs $395 annually, and a professional subscription to UpToDate costs $499 annually. Institutions must contact the vendor for pricing; however, based on the author’s own experience, generally the institutional cost of EEP is significantly lower than other point-of-care tools. EEP offers free 30-day trials for both types of subscribers.

EEP was included in a 2016 study that Johnson, Emani, and Ren conducted evaluating six mobile point-of-care tools on how effectively they were able to meet the clinical information needs of internal medicine clinicians. The tools were examined for breadth of content, usability, and quality. The authors found that all of the tools reviewed were relatively similar across these dimensions, but the mobile version of EEP scored somewhat lower than the other tools on usability [4].

## SUMMARY

EEP is a robust, cost-effective, and efficient tool for providing clinicians with current information to facilitate evidence-based practice. The credentials of the editors and the Medical Advisory Board suggest that it is a resource that is carefully vetted by medical experts, so the information is most likely highly reliable. While there are a number of other point-of-care clinical tools available on the market, EEP’s special features such as the daily POEM alerts, the clearly labeled levels of evidence, and the Drug Safety Alerts set this database apart. The low cost as well as the similarity to other point-of-care tools in terms of content and quality make it an appealing option to those with limited budgets.
